# Advancing the diagnosis and classification of renal cell carcinomas

**DOI:** 10.1186/s12916-021-02105-2

**Published:** 2021-09-06

**Authors:** Joseph A. Rothwell

**Affiliations:** Université Paris-Saclay, UVSQ, Inserm, Gustave Roussy, Exposome and Heredity team (CESP U1018), Villejuif, F-94805 France

**Keywords:** Renal neoplasia, Proteomics, Total protein approach, Renal cancer diagnosis

## Background

Renal cancer is of concern due to its rising incidence worldwide, with new cases per year expected to climb by as much as 20% by 2030 [1]. Renal neoplasms encompass a variety of malignant and benign tumours with varying prognoses. Accurate classification and diagnosis currently rely on immunohistochemistry to detect known protein markers, in combination with examination of morphological characteristics. Accurate identifications are not always possible, which in turn hinders the delivery of a diagnosis and process to adapted treatment plans. Advanced-stage renal cell carcinomas carry extremely poor prognoses, so improved methods are urgently needed.

High-resolution mass spectrometry (MS) is the cornerstone of proteomics technologies and rapidly evolving tool that promises to drive such methods forward. In MS-based proteomics, large numbers of proteins can be characterised and their absolute amounts measured in solid and liquid biopsies, termed the “total protein approach” (TPA) [2]. Tumours may then be distinguished by the unique concentration ranges of protein biomarkers. With an unparalleled depth of interrogation of tumour proteomes, the technique enables new protein markers of specific neoplasms to be found among the vast number of overexpressed proteins in tumour cells. TPA does not require labelling of the sample or the addition of specific antibodies to the sample, and neither are calibration standards required. While some studies have applied the technique to cell or animal models, only one has been conducted with tissue biopsies from cancer patients [3], whose aim was to elucidate changes in energy metabolism and plasma membrane transport, rather than improve clinical practice and patient outcomes. No previous study has applied TPA to renal neoplasms.

## New data

In the new study by Jorge et al. [4], TPA is applied, for the first time, to the diagnosis of renal cell carcinomas. The investigators collected human renal tissue biopsies of malignant clear cell renal cell carcinoma, papillary renal cell carcinoma and chromophobe renal cell carcinoma, as well as the benign renal oncocytoma. Normal adjacent tissue samples were used as a control. It is hypothesised that due to extensive molecular heterogeneity between these tumour types [5], the depth of profiling offered by TPA will allow substantial improvements in classification accuracy over current methods, via the discovery of sensitive and specific biomarker panels.

Proteins were first extracted from 27 tumour biopsies and controls by trypsin digestion and these extracts injected into a liquid chromatography—a mass spectrometry instrument. A total of 1234 proteins were reliably detected in more than one sample type, and these were retained for quantification. Proteomes varied the most between tumour biopsies, while normal adjacent tissue samples were the most homogenous. Statistical analysis identified 205 proteins whose abundances distinguished the different tumour subtypes, of which some were already known as immunohistochemical markers for specific neoplasms. Smaller panels of proteins were then chosen that distinguished each neoplasm from all others and normal tissue. The most discriminant protein for each neoplasm was validated on tissue microarrays in an immunohistochemical analysis of 128 separate renal cell carcinoma and control tissue biopsies. The investigators were able to assess the efficacy of protein markers for each of the malignant renal carcinomas by determining the proportion of cells stained in each tissue type for each protein. PLIN2 was thus proposed as a sensitive and specific marker of clear cell renal cell carcinoma, while beta-tubulin III, diffuse LAMP1 and HK1 were proposed as efficient markers of papillary renal cell carcinoma, chromophobe renal cell carcinoma and renal oncocytoma, respectively.

The findings were further strengthened by their biological plausibility. Of the 81 discriminant proteins for clear cell renal cell carcinoma, 46 had been described previously, of which the authors highlight thymidine phosphorylase and PLIN2 as mediators of cell proliferation and the inhibition of apoptosis. The similarity of renal carcinoma proteomes to each other was consistent with their cellular origins, either cells of the proximal tubules or intercalated cells of the distal nephron and collecting ducts. Moreover, the benign renal oncocytoma proteome was most similar to that of normal adjacent tissue, lending additional confidence in the context of deployment in clinical settings.

## Impact and implications

Proteomics analyses have previously been applied to tumour classification using gel-based protein quantification, including for renal cell carcinomas [6], but the study of Jorge et al. makes a convincing case that MS-based TPA will be able to offer superior speed and accuracy. Some barriers will need to be overcome before proteomics, and TPA will be able to offer an alternative to immunohistochemistry in clinical settings. Firstly, MS-based platforms for proteomic analysis remain costly to establish. Secondly, although suitable instruments are becoming more affordable, reference databases of tumour TPA concentrations will be required and are not currently available. In the meantime, TPA will undoubtedly develop as a valuable method of discovering new protein biomarkers for use in existing immunohistochemistry diagnosis. Further on, given the minimal sample preparation required and potential for accurate high-throughput analyses, the authors conclude that it could potentially become a standard diagnostic tool, aiding accurate prognoses and guiding treatment plans for advanced stage renal carcinomas. A limitation of the study of Jorge et al. is the low number of biopsies per neoplasm analysed in the study, given the known inter-tumour and intra-tumour variability in renal neoplasms [7]. Future validations will aim to scale up analyses to larger patient cohorts.

## Data Availability

Not applicable.
